# Point of Care Ultrasound Used to Diagnose Nontyphoidal Endocarditis

**DOI:** 10.24908/pocus.v9i1.16937

**Published:** 2024-04-22

**Authors:** Zahraa Y Alqallaf, Ossama S Maadarani, Mohamed E Elhabibi, Mohamad Abdelfatah, Zouheir I Bitar

**Affiliations:** 1 BSc in Biology student, Dalhousie University Halifax, NS Canada; 2 Critical Care Unit, Ahmadi Hospital Ahmadi Kuwait; 3 Kuwait Oil Company Ahmadi Kuwait

**Keywords:** POCUS, Endocarditis, Non-typhoidal Disease

## Abstract

Point of care ultrasound (POCUS) can make an expedited diagnosis, which might lead to early correct management. POCUS should be used in a systemic and integrated approach to evaluate multiple organs in patients with sepsis and septic shock. We present a rare case of sepsis due to nontyphoidal Salmonella endocarditis with splenic abscess in which a multiorgan POCUS examination led to expedited treatment.

## Introduction

Incorporating point of care ultrasound (POCUS) has become the standard care in evaluating critically ill patients. Many adopted protocols for different critical situations have been validated 1. POCUS facilitates rapid diagnosis, which can expedite management. Sepsis and septic shock are emergencies in which early recognition can lead to improved outcomes 2. The POCUS exam for sepsis or septic shock should be systematic and integrate the assessment of multiple organ systems 3. This comprehensive approach improves the provider’s ability to diagnose the presence of sepsis, identify the culprit infection, and narrow the differential diagnosis 3. The initial clinical presentation can be nonspecific in patients with sepsis and septic shock 4. The diagnosis of sepsis and septic shock requires clinical examination, laboratory results, radiologic tests, and microbiologic data 4. In acute situations, advanced imaging modalities, such as computed tomography or magnetic resonance imaging, may be difficult to access because of the instability of patients.

POCUS is a clinician-performed bedside modality that can help diagnose sepsis and detect the source of sepsis during the assessment of critically ill patients 5. POCUS examination in sepsis should be systemic and comprehensive in order to narrow the differential diagnosis 5.

Only 5% of infected patients with nontyphoidal Salmonella gastrointestinal illness might develop bacteremia 6. Immunocompromised patients and patients with diabetes are more likely to develop bacteremia from nontyphoidal Salmonella 6. In patients with nontyphoidal Salmonella bacteremia, 25% might develop arteritis or endocarditis, especially patients over 50 years-old 7. The global incidence of bloodstream infection with nontyphoidal Salmonella has been estimated at 50 cases per 100,000, with Africa being the most affected 8. We present a case of sepsis in which POCUS helped determine the source of sepsis and expedited early treatment.

## Case Presentation

A 60-year-old woman with ischemic heart disease, type II diabetes mellitus, hypertension, and chronic kidney disease presented to the emergency department with two weeks of fever, crampy left sided abdominal pain, and irritability. She had been diagnosed with ischemic heart disease and ST -elevation myocardial infarction, followed by coronary artery bypass surgery ten years prior. The results of echocardiography after surgery were normal. Since the age of 30, she has had poorly controlled diabetes mellitus (HbA1c of 11.7), which has been complicated by diabetic nephropathy, diabetic retinopathy, and multiple vitreous hemorrhages in the left eye. She had been given oral antibiotics from her family physician one week before admission. She reported no nausea, vomiting, diarrhea, or constipation. Examination of the patient showed temperature 38.7 °C, heart rate 110 beats per minute, blood pressure 90/45 mmHg, respiratory rate 24 breaths per minute, and oxygen saturation 99% on room air. 

**Figure 1:**
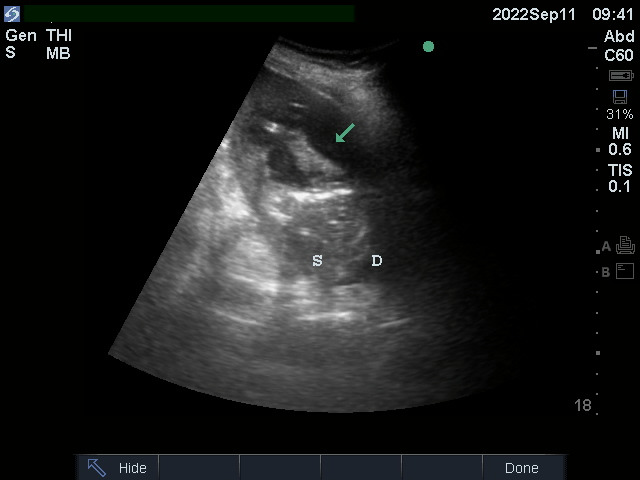
Abdominal POCUS with multiple hypoechoic areas (arrow) in the spleen (S). D is the diaphragm.

**Figure 2:**
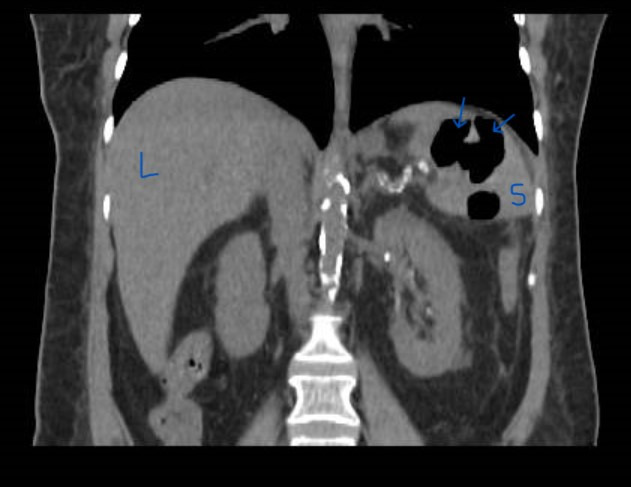
CT with intravenous contrast revealedlarge area of hypoattenuation within the spleen, mainly in the peripheral region that was suggestive of splenic infarction with signs of splenic abscess. L liver, S spleen.

The patient was conscious, and chest sounds were normal, with a heart grade 2/6 systolic murmur at the right upper sternal border with radiation to the carotid arteries. Her abdomen was tender on the left upper quadrant. The laboratory test results are shown in Table 1. There was elevated procalcitonin, leukocytosis (mainly neutrophils), and high C-reactive protein. The electrocardiogram revealed normal sinus rhythm. Abdominal POCUS showed splenomegaly with multiple hypoechoic areas measuring a few mm to 6 cm in diameter (video S1, Figure 1). The kidneys appeared normal and the inferior vena cava collapsed. Cardiac POCUS showed a normal-size hyperdynamic left ventricle. There was aortic sclerosis and a mobile mass attached to the right coronary cusp and left coronary cusp (LCC) with possible vegetation with mild aortic regurgitation (video S2). Chest POCUS showed bilateral A-lines and no evidence of pleural effusion. Abdominal computed tomography with intravenous contrast showed significant splenomegaly and a large area of hypoattenuation within the spleen, mainly in the peripheral region that was suggestive of splenic infarction with signs of splenic abscess (Figure 2). The patient was diagnosed with septic shock, aortic valve endocarditis, splenic infarction, and abscess. The patient then underwent ultrasound-guided drainage of the splenic abscess which grew Salmonella species sensitive to ceftriaxone. The blood culture was negative, likely because the patient received oral cephalosporin before admission to the hospital. The serology for Coxiella burnetii, Bartonella spp., Chlamydia spp., and Brucella was negative.

**Table 1:** Laboratory values on admission.

**Variable**	**Results (On admission)**	**3-Days after admission**	**Normal Range**
Sodium (mmo/L)	146	139	136–145
Potassium (mmo/L)	5	4	3.8-5.2
Urea nitrogen	8	6	3.5-6.1
Creatinine	87	90	45-110
Lactate	3	1.2	< 1.2
White cell count (per mm^3^)	21	15	4,500–11,000
Neutrophils (%)	83	70	48-75
Hemoglobin g/dL	9.4	10	13.5–16.5
Platelet (per mm^3^)	390	500	150,000–350,000
International normalized ratio	1.21	1.1	0.8 -1.1
Procalcitonin (ng/ml)	57	11	< 0.01
C-reactive protein (mg/L)	230	180	< 20
Glucose (mmol/L)	14	8	4.5-6.2

The patient completed 45 days of ceftriaxone and two weeks of gentamycin with improvement. After one month of follow-up, the patient was stable and afebrile, and repeat imaging showed resolution of the splenic abscess and aortic vegetation.

## Discussion

The patient met one major clinical criterion (echocardiography, new regurgitation, and vegetation) and three minor clinical criteria (fever, splenic infarction, and abscess, positive culture for an organism involved in infective endocarditis from a sterile body site other than cardiac tissue, cardiac prosthesis, or embolus) of the 2023 Duke–ISCVID Criteria for Infective Endocarditis, which led to a diagnosis of infective endocarditis 9.

The negative blood culture in the present case was likely due to antimicrobial treatment received prior to admission. Other causes of culture-negative endocarditis are microorganisms with demanding growth characteristics in vitro (such as Gemella or Granulicatella, intracellular bacteria that cannot be cultured from blood using standard microbiologic testing methods) 10. In the present case, the organism was isolated from tissue splenic culture.

The International Collaboration on Endocarditis reported non-HACEK gram-negative bacteria in 49 of 2761 (1.8%) infective endocarditis cases 11.

The management guidelines for non-HACEK gram-negative aerobic bacilli include early surgery and long-term (at least six weeks) antimicrobial drugs 12. The suggested antimicrobials were beta-lactam and aminoglycoside addition of quinolones. The reported current patient did well with ceftriaxone and aminoglycoside without surgery 12.

POCUS protocols are well-designated for shock and hypoxic respiratory failure, but there is no specific ultrasound protocol for sepsis and septic shock 1, 5. The RUSH protocol differentiates different types of shock, including distributive shock, which could be due to sepsis 1. Apart from the early diagnosis of septic shock, the identification and effective source control of sepsis and the rapid implementation of resuscitative measures have a positive impact on the outcome of the disease 13. POCUS can aid in resuscitation measures, is associated with improved clinical outcomes in patients with shock, and helps improve the safety of bedside procedures 3.

## Conclusion

We report a rare case of nontyphoidal endocarditis with splenic infarction and abscess diagnosed by POCUS. This case illustrates the potential benefit of multiorgan POCUS in the evaluation of patients with sepsis.

## Consent

The patient consented and permitted to publish his/her clinical history.

## Conflicts of Interests

The authors declare there are no competing interests.

## Supplementary Material

 Video S1 Abdominal POCUS showed splenomegaly with multiple hypoechoic areas measuring a few mm to 6 cm in diameter.

Video S2 There was aortic sclerosis and a mobile mass attached to the right coronary cusp and left coronary cusp with possible vegetation with mild aortic regurgitation.
